# INTESTINAL ENDOMETRIOSIS: OUTCOMES FROM A MULTIDISCIPLINARY SPECIALIZED REFERRAL CENTER

**DOI:** 10.1590/0102-6720202400013e1806

**Published:** 2024-07-01

**Authors:** Leandro Cardoso BARCHI, Gustavo Yano CALLADO, Rogério Bonassi MACHADO, Marcelo Antunes CHICO, Daniella Closer DAMICO, Daniela Pereira LACERDA, Rocco RICCIARDI, Rodrigo Moises de Almeida LEITE

**Affiliations:** 1 São Leopoldo Mandic, Faculty of Medicine, Campinas (SP), Brazil;; 2 Gastromed Instituto Zilberstein, São Paulo (SP), Brazil;; 3 São Luiz Rede D’or, Hospital Osasco Endometriosis Centre, São Paulo (SP), Brazil;; 4 Faculdade Israelita de Ciências da Saúde Albert Einstein, Hospital Israelita Albert Einstein, São Paulo (SP), Brazil;; 5 Harvard Medical School, Massachusetts General Hospital, Boston (MA), USA;; 6 Faculdade de Medicine de Jundiai, Jundiaí (SP), Brazil.

**Keywords:** Endometriosis, Intestines, Abdominal pain, Sigmoid colon, Rectum, Endometriose, Intestinos, Dor abdominal, Colo sigmoide, Reto

## Abstract

**BACKGROUND:**

Deep penetrating endometriosis (DE) can affect abdominal and pelvic organs like the bowel and bladder, requiring treatment to alleviate symptoms.

**AIMS:**

To study and investigate clinical and surgical outcomes in patients diagnosed with DE involving the intestines, aiming to analyze the effectiveness of surgical treatments.

**METHODS:**

All cases treated from January 2021 to July 2023 were included, focusing on patients aged 18 years or older with the disease affecting the intestines. Patients without intestinal involvement and those with less than six months of post-surgery follow-up were excluded. Intestinal involvement was defined as direct invasion of the intestinal wall or requiring adhesion lysis for complete resection. Primary outcomes were adhesion lysis, rectal shaving, disc excision (no-colectomy group), and segmental resection (colectomy group) along with surgical complications like anastomotic leak and fistulas, monitored for up to 30 days.

**RESULTS:**

Out of 169 patients with DE surgically treated, 76 met the inclusion criteria. No colectomy treatment was selected for 50 (65.7%) patients, while 26 (34.2%) underwent rectosigmoidectomy (RTS). Diarrhea during menstruation was the most prevalent symptom in the RTS group (19.2 vs. 6%, p<0.001). Surgical outcomes indicated longer operative times and hospital stays for the segmental resection group, respectively 186.5 vs. 104 min (p<0.001) and 4 vs. 2 days, (p<0.001). Severe complications (Clavien-Dindo ≥3) had an overall prevalence of 6 (7.9%) cases, without any difference between the groups. There was no mortality reported. Larger lesions and specific symptoms like dyschezia and rectal bleeding were associated with a higher likelihood of RTS. Bayesian regression highlighted diarrhea close to menstruation as a strong predictor of segmental resection.

**CONCLUSIONS:**

In patients with DE involving the intestines, symptoms such as dyschezia, rectal bleeding, and menstrual period-related diarrhea predict RTS. However, severe complication rates did not differ significantly between the segmental resection group and no-colectomy group.

## RESUMO

**RACIONAL:**

A endometriose profunda infiltrativa (EP) pode afetar órgãos abdominais e pélvicos, tais como o intestino e a bexiga, necessitando de tratamento para aliviar os sintomas.

**OBJETIVOS:**

Estudar e investigar resultados clínicos e cirúrgicos em pacientes com diagnóstico de EP envolvendo o intestino, visando analisar a eficácia dos tratamentos cirúrgicos.

**MÉTODOS:**

Foram incluídos todos os casos atendidos de janeiro de 2021 a julho de 2023, com foco em pacientes com 18 anos ou mais com a doença acometendo o intestino. Foram excluídos pacientes sem comprometimento intestinal e aqueles com menos de seis meses de acompanhamento pós-operatório. O envolvimento intestinal foi definido como invasão direta da parede intestinal ou necessidade de lise de aderências para ressecção completa. Os desfechos primários foram lise de aderências, raspagem retal e excisão discóide (grupo não colectomia), e ressecção segmentar (grupo colectomia), juntamente com complicações cirúrgicas como fístulas, monitoradas por até 30 dias.

**RESULTADOS:**

Das 169 pacientes com EP tratadas cirurgicamente, 76 preencheram os critérios de inclusão. Não foi indicado colectomia em 50 (65,7%) pacientes, enquanto 26 (34,2%) foram submetidos à retossigmoidectomia (RTS). A diarreia durante a menstruação foi o sintoma mais prevalente no grupo RTS (19,2% vs. 6,0%, p<0,001). Os resultados cirúrgicos indicaram tempos operatórios e internações hospitalares mais longos para o grupo de ressecção segmentar, respectivamente, 186,5 vs. 104 min (p<0,001) e 4 vs. 2 dias, (p<0,001). As complicações graves (Clavien-Dindo ≥3) tiveram prevalência global de 6 (7,9%) casos, sem diferença entre os grupos. Não houve mortalidade relatada. Lesões mais graves e sintomas específicos como disquezia e sangramento retal foram associados a maior probabilidade de indicação de RTS. A regressão bayesiana destacou a diarreia próxima à menstruação como um forte preditor de ressecção segmentar.

**CONCLUSÕES:**

Em pacientes com endometriose envolvendo os intestinos, sintomas como disquezia, sangramento retal e diarreia relacionada ao período menstrual predizem a indicação de RTS. No entanto, as taxas de complicações graves não diferiram significativamente entre o grupo de ressecção segmentar e o grupo sem colectomia.

## INTRODUCTION

Endometriosis is defined as the presence of endometrial-like tissue, estrogen dependent outside the uterus that induces a chronic inflammatory reaction. It is the second most common benign gynecological condition in women of reproductive age, affecting around 7–15% of the female population. The true pathogenesis of endometriosis remains unclear. The most accepted is the retrograde menstruation reflux hypothesis. Other hypotheses are the theories of celomic metaplasia, the trafficking of stem cells, and the embryonic rests which have also been proposed and are under investigation. All these possibilities only confirm that endometriosis is a complex disease and probably of multifactorial origin^29^.

When the endometrial tissue is found more than 5 mm below the peritoneal surface it is called deep endometriosis (DE). The prevalence is estimated at around 5–12% in women with pelvic endometriosis. The DE may affect the bowel, bladder, ureters, pelvic nerves, omentum, and diaphragm, and even translocate to the chest^22^. The most frequent site of extragenital endometriosis is along the bowel, more specifically in the upper rectum (90% of the cases), in contiguity with lesions that influence the uterus, but it can be present anywhere along the lower gastrointestinal tract. The implants usually affect the serous layer, but eventually may manifest as deeply infiltrative lesions of the muscularis and more rarely the mucosa layer, causing retractile thickening and fibrosis of the bowel wall. These endometrial implants can be identified by endoscopic methods. The estimated incidence of colorectal endometriosis in patients with DE varies between 5–38%. At least half of patients with rectal lesions develop a second intestinal lesion^12,18^.

A long diagnostic delay after symptom onset is common. The main gynecological symptoms are dysmenorrhea, pelvic pain, deep dyspareunia, and infertility^25^. Patients with intestinal endometriosis present with symptoms including diarrhea, constipation, tenesmus, dyschezia, and rectal bleeding. Symptoms are usually synchronous with menstruation, but they can occur apart from the menstrual period. Patients suspected of having endometriosis should have a thorough physical examination and complementary diagnostic tests, such as transvaginal ultrasonography (TVUS) performed after bowel preparation, pelvic and upper magnetic resonance (MRI), endorectal ultrasound, and colonoscopy^20^.

The goals of the treatment of DE are to recover fertility, relieve symptoms, and improve quality of life, while preventing possible complications such as intestinal obstruction^9^. Medical management including nonsteroidal anti-inflammatory drugs, oral contraceptives, progesterone, and gonadotropin-releasing hormone analogs has some effectiveness and may be advisable for those who are not surgical candidates or who prefer to avoid surgery. However, patients with DE may require surgical treatment when symptoms exacerbate. Surgical treatment is an option after failure of medical treatment, in case of progressive lesions, or in case of patients with impaired sexual and/or reproductive functions. It is currently considered the first option in symptomatic patients with invasive intestinal compromise, as it leads to lasting relief of symptoms and improvement in quality of life^3^.

Several minimally invasive approaches have been described to treat DE with intestinal involvement (bowel resection, disc excision and intestinal shaving). The aim of this study was to identify the characteristics of patients with DE with intestinal involvement, the type of intestinal resection performed, as well as the main outcomes, and the risk factors for complications in a multidisciplinary specialized referral center.

## METHODS

In January 2021, the Endometriosis Center at Hospital São Luiz Rede D’or Osasco was created with the aim of treating patients with deep endometriosis. The center is composed of two gynecologists, a gastrointestinal surgeon, a radiologist, a nurse, a psychologist, and a dietician. All cases included were conducted by the same surgical team, in a tertiary, specialized hospital between January 2021 and July 2023.

### Cohort abstraction

This is a retrospective study of consecutive cases of DE patients with intestinal involvement treated in a single specialized center. All clinical data came from our prospectively collected database. All cases operated between January 2021 and July 2023 were considered. Inclusion criteria comprised patients 18 years and older and diagnosed with DE with intestinal involvement. Patients without intestinal involvement and with less than 6-month follow-up after surgery were excluded from the analysis. The intestinal involvement was considered whenever there was a direct invasion of the intestinal wall, or at least, an adhesion lysis was necessary to accomplish the complete resection.

Preoperative evaluation included laboratorial tests, TVUS, upper abdominal and pelvic MRI, and colonoscopy in all cases. Endorectal ultrasound was performed in selected cases. All patients received nutritional and psychological support.

Patients were classified by the American Anesthesiology Association (ASA) scoring system^21^. Perioperative complications were described according to the Clavien-Dindo classification. Clavien-Dindo complications ≥3 were considered severe^14^. Surgical mortality was considered when death occurred within 90 days of surgery.

All patients underwent retrograde bowel preparation with enemas the day before surgery. Before anesthetic induction, antibiotic prophylaxis with intravenous Cefazolin was given. When segmental resection was necessary, a small Pfannenstiel incision was performed to remove the surgical specimen. The extent of intestinal resection (rectal or colonic resection, disc excision, rectal shaving, enterectomy, and/or appendectomy) was based on the location of the disease to obtain free margins. Mechanical anastomosis with a 31- or 33-mm circular stapler was used in patients submitted to rectal segmental resection or disc excision. All patients were operated by laparoscopy.

Patients undergoing no-colectomy surgery received a liquid oral diet on the same day of the procedure. Those who underwent RTS received the diet on the first postoperative day.

The predictors analyzed included gynecological and any intestinal symptoms, onset of symptoms before diagnosis, hospital length of stay, 30-day hospital readmission, operative time, pregnancy history, and previous endometriosis treatments. The type of gynecological surgery performed, the type of intestinal resection, and the size of the intestinal lesions removed were also analyzed.

The main outcomes of interest were the performance of RTS vs. no-colectomy surgery (including adhesion lysis, rectal shaving, and disc excision), and the incidence of surgical complications (including anastomotic leak, fistulas, vaginal wall dehiscence, or ureter lesion). All outcomes were censored at 30 days.

The study was approved by the Hospital Ethics Committee and registered in the “Plataforma Brasil” under Certificate of Presentation for Ethical Appreciation (CAAE) 75922723.8.0000.5374.

#### Statistical analysis

Two sample t-tests and proportion tests were applied to analyze demographic differences between the two groups. Bayesian model averaging for linear regression was utilized to build a variable inclusion map exploring the importance of predictors for surgical complications and RTS. Chi-squared tests and linear regression were used to estimate unadjusted risk ratios and coefficients (coef) for the outcomes of interest. Adjusted risk ratios (aRR) were obtained through Poisson regression after adjusting for age, previous clinical conditions, ASA score, previous operation for endometriosis, and duration of symptoms. Multiple linear regression model was used to obtain coefficients to analyze the correlation between the duration of symptoms and outcomes of interest. The analysis was conducted on Stata Statistics version 18 Standard Edition.

## RESULTS

### Demographics

One hundred and sixty-nine DE patients were surgically treated during the study period. Of these, 93 did not meet the inclusion criteria (no intestinal involvement) and were excluded from the analysis. Our final cohort consisted of 76 female patients. The median age was 39.8 years (range 24–50). The median duration of symptoms onset until final diagnosis was 20 months (range 0–240).

The final analysis was performed by dividing our cohort between patients who were treated without colectomy (including only adhesion lysis, rectal shaving, or disc excision) versus patients who underwent segmental resection (RTS cases). The no-colectomy cohort consisted of 50 (65.7%) patients, while 26 (34.2%) underwent RTS.

The most common gynecological symptom was pelvic pain in 52 (68.4%) patients, followed by dysmenorrhea affecting 49 (64.4%), and dyspareunia in 37 (48.6%). Pelvic pain was also the most frequent symptom in the no-colectomy group (74.0% vs. 61.5%; p=0.020; p<0.050). Twenty-five patients (32.8%) reported previous medical treatment for endometriosis (including oral progestin-based contraceptives, hormonal intrauterine devices, or long-acting protein implants). Sixteen patients (21%) had at least one previous surgery for endometriosis.

### Intestinal symptoms

Intestinal symptoms were present in 25 (32.8%) patients. The most common symptoms observed were constipation in 14 (18.4%) patients, followed by dyschezia in 11 (14.4%), and diarrhea close to the menstrual period in 8 (10.5%). The presence of diarrhea during the menstrual period was statistically significantly more prevalent in the segmental resection group (19.2% vs. 6.0%; p<0.001). The clinical characteristics of all patients are summarized in Table 1.

**Table 1:** Demographic and clinical characteristics in the cohort of 76 patients.

Variables	All patients	No-colectomy group	Segmental resection group	p-value
n (%)	76 (100)	50 (65.8)	26 (34.2)	
Age (years) mean (IQR) or (range)	39.8 (34–43) (24–50)	39 (7.8) (24–59)	40.5 (5.8) (30–55)	0.386
Duration of symptoms (months)	20 (12–24) (0–240)	33 (43.3) (3–240)	30 (28.3) (0–120)	0.067
Dysmenorrhea	49 (64.4)	32 (64)	17 (65.3)	0.868
Dyspareunia	37 (48.6)	23 (46)	14 (53.8)	0.281
Pelvic pain	52 (68.4)	37 (74)	16 (61.5)	0.020
Infertility	15 (19.7)	12 (24)	3 (11.5)	0.200
Abnormal uterine bleed	13 (23.2)	8 (16)	5 (19.2)	0.727
C-section (1 or more)	32 (42.1)	19 (38)	13 (50.0)	0.628
Pregnancy history (1 or more)	39 (51.3)	26 (52)	13 (50.0)	0.670
Abortion (1 or more)	6 (7.8)	6 (12)	0	0.065
Back pain	5 (6.5)	3 (6)	2 (7.6)	0.670
Dyschezia	11 (14.4)	5 (10)	6 (23.0)	0.128
Tenesmus	2 (2.6)	2 (4)	0	0.308
Constipation	14 (18.4)	11 (22)	3 (11.5)	0.211
Diarrhea	8 (10.5)	3 (6)	5 (19.2)	<0.001
Hematochezia	1 (1.3)	0	1 (3.8)	0.167
ASA score 1	51 (67.1)	35 (70)	16 (61.5)	0.463
ASA score 2	25 (32.8)	15 (30)	10 (38.4)	0.463
Previous surgery for endometriosis	16 (21.0)	11 (22)	5 (19.2)	0.378
Previous medical treatment	25 (32.8)	18 (36)	7 (26.9)	0.182

Data given in median (IQR: interquartile range) or n (%); ASA: American Society of Anesthesiologists.

### Surgical outcomes

The overall median operative time was 133.5 min (range 45–401). The overall median hospital length of stay was 2 days (range 1–9). The operative time and length of hospital stay were longer in the segmental resection group when compared to the no-colectomy group, respectively, 186.5 min (range 115–305) vs. 104 min (range 45–401), p<0.001, and 4 days (range 2–6) vs. 2 days (range 1–9), p<0.001.

In 3 (3.9%) patients more than one type of resection was necessary (disc excision + shaving). Enterectomy was performed in 5 (6.5%) and appendectomy in 14 (18.4%). A diverting ileostomy was necessary in one (1.3%) patient due to stapling failure.

Thirty-one patients (40.7%) underwent concurrent hysterectomy. Performing hysterectomy was significantly more prevalent in the no-colectomy group (48.0% vs. 30.7%; p<0.001). The presence of retrocervical/uterine torus nodes had an overall prevalence in 53 (69.7%) patients, being more frequently observed in the no-colectomy group (74.0% vs. 61.5%; p=0.020).

Thorough distal ureter dissection to complete removal of all lesions was necessary in 14 (18.4%) patients. Of those, the left ureter was the most affected (71.4%). In one patient there was a direct invasion of the left ureter and a ureter resection with primary anastomosis was performed. Although there was no complication regarding the anastomosis, two cystoscopies were necessary for repositioning the double-J stent. Twenty-four (31.5%) patients had nodules in the vesicouterine recess. In 7 (36.8%) of those, there was direct invasion of the bladder wall.

Hospital readmission was necessary for 8 (10.5%) patients. The overall morbidity rate was 31.6% (24 patients). Severe complications (Clavien-Dindo ≥3) occurred in 6 cases (7.9%), and other surgical morbidity in 5 (6.5%). Reoperation was necessary in 2 (2.6%) patients (new vaginal dome suturing). One of these patients developed pulmonary thromboembolism and presented with vaginal bleeding after full anticoagulation therapy. There were 2 (2.6%) cases of fistulas treated conservatively (one rectovaginal and another ureterovaginal). Finally, one patient developed sepsis due to urinary tract infection. There were no conversions to open surgery and no 90-day mortality. Surgical outcomes are presented in Table 2.

**Table 2:** Surgical outcomes in the cohort of 76 patients with endometriosis.

Variables	All patients	Segmental resection group	No-colectomy group	p-value
n (%)	76 (100)	26 (34.2)	50 (65.8)	
Operative time (minutes) (range)	133 (45–401)	186.5 (115–305)	104 (45–401)	<0.001
In hospital stay (days)	2 (1–9)	4 (2–6)	2 (1–9)	<0.001
Enterectomy	5 (6.5)	0	5 (10)	>0.050
Appendectomy	14 (18.4)	4 (15.3)	10 (20)	0.296
Omentum	1 (1.3)	1 (1.3)	0	>0.050
Diaphragm	1 (1.3)	1 (1.3)	0	>0.050
Hysterectomy	32 (42.1)	8 (30.7)	24 (48)	<0.001
Right ovarian	22 (28.9)	7 (26.9)	15 (30)	0.385
Left ovarian	28 (26.8)	9 (34.6)	19 (38)	0.614
Retrocervical/uterine torus	53 (69.7)	16 (61.5)	37 (74)	0.020
Vaginal dome	6 (7.8)	1 (3.8)	5 (10)	>0.050
Vesicouterine recess	24 (31.5)	9 (34.6)	15 (30)	0.639
Bladder	7 (9.2)	1 (3.8)	6 (12)	>0.050
Left ureter region	10 (13.1)	5 (19.2)	5 (10)	0.112
Right ureter region	4 (5.2)	2 (7.6)	2 (4)	0.370
Ureter resection	1 (1.3)	1 (2.0)	0	>0.050
Left inferior hypogastric plexus	4 (5.2)	2 (7.6)	2 (4)	0.370
Right inferior hypogastric plexus	1 (1.3)	1 (3.8)	0	>0.050
Ileostomy	1 (1.3)	0	1 (2)	>0.050
30-day hospital readmission	8 (10.5)	3 (11.5)	5 (10)	0.892
Clavien-Dindo 1 and 2	18 (23.6)	7 (26.9)	11 (22)	0.686
Clavien-Dindo 3 and 4	6 (7.9)	2 (7.6)	4 (8)	0.898
Reoperation	2 (2.6)	0	2 (4)	>0.050

### Size and characteristics of intestinal endometriosis lesions

In the 26 patients who underwent RTS, the median size of the largest diameter of intestinal lesions was 3.1 cm (range 1–5). Four (15.3%) patients had two different lesions in the same surgical specimen. In 9 (34.6%) patients, submucosal layer invasion was present. In the 5 (6.5%) patients submitted to disc excision, the median size of the largest diameter lesions was 2.6 cm (range 2.0–3.3). Thirty-one (40.7%) patients underwent rectal shaving, and the median size of the largest diameter lesions was 1.6 cm (range 0.5–4.0). Four (13.3%) patients received two shaving resections in different locations. Appendectomy was performed in 14 patients. Of those, 5 (35.7%) had no endometriosis lesions found. Larger lesions were more frequent in patients treated with RTS when compared to rectal shaving and disc excision, respectively (3.1 cm vs. 1.6 cm vs. 2.6 cm, p<0.001). The size of the lesion was not associated with increase in the incidence of surgical complications (coef 0.025; 95%CI -2.36 +2.41; p=0.983). The main characteristics of intestinal lesions are represented in Table 3.

**Table 3:** Intestinal endometriosis lesions size.

	RTS		Rectal shaving		Disc excision	p-value
n (%)	26 (34.2)		31 (40.7)		5 (6.5)	
Median Size (cm) (IQR) (range)	3.1 (2.0–3.3) 1–5		1.6 (1– 2.5) (0.5–4.0)		2.6 (2.5–3.1) (2–3.3)	<0.001
	**Univariate regression (95%CI)**	**p-value**	**Multivariate regression (95%CI)**	**p-value**		
Major complications vs. Lesion size	Coef 0.005 (-0.09 +0.10)	0.913	Coef 0.025 (-2.36 +2.41)	0.983		

Data given in median (IQR: interquartile range) or n (%); CI: confidence interval; RTS: rectosigmoidectomy; Coef: coefficient.

### Bayesian regression and predictor inclusion map

**Figure 1:**
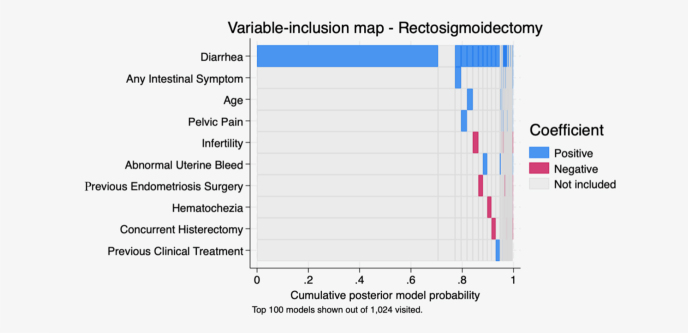
Visual map with Bayesian regression model demonstrating positive and negative correlations with rectosigmoidectomy. No strong single predictor for major surgical complications could be identified.

**Figure 2:**
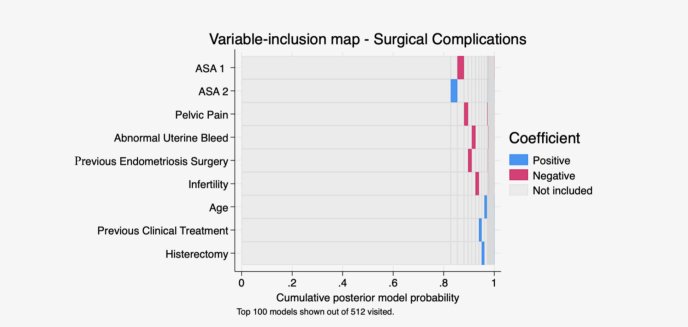
Visual map with Bayesian regression model demonstrating positive and negative correlations with surgical complications. Diarrhea in the perimenstrual period was the strongest predictor for rectosigmoidectomy.

We performed Bayesian regression with linear regression with Stata built-in command to construct a visual map exploring the importance of predictors of RTS (Figure 1) or surgical complications (Figure 2). These models allow for visual screening by demonstrating positive and negative correlations in a logarithmic scale of relevant predictors to include in our regression model. The results revealed that diarrhea in the perimenstrual period was the strongest predictor for RTS, whereas no single predictor for major surgical complications could be identified.

### Risk factors for rectosigmoidectomy and major complications

The clinical features associated with higher likelihood of RTS included dyschezia (aRR 1.77; 95%CI 1.20–3.39; p=0.014; p<0.050), rectal bleeding (aRR 3.0; 95%CI 2.17–4.13; p=0.016; p<0.050), and diarrhea (odds ratio [OR] 1.99; 95%CI 1.03–3.82; p=0.038; p<0.050). There was no significant association between RTS and constipation (aRR 0.62; 95%CI 0.17–2.19; p=0.419; p>0.050) or duration of symptoms (coef 0.004; 95%CI -0.003 +0.022; p=0.719; p>0.050). The composite outcome of any intestinal symptom was not associated with a higher rate of RTS (aRR 1.29; 95%CI 0.68–2.45; p=0.432; p>0.050). Still, the no-colectomy surgery group was found to be an independent risk factor for reoperation (0.29; 95%CI 0.12–0.70; p<0.001). All data are presented in Table 4.

**Table 4:** Risk factors for segmental resection and surgical complications.

n (%)	Number of events	Segmental resection 26 (34.2)		No-colectomy surgery 50 (65.7)	
		Univariate risk ratio (95%CI)	p-value	Multivariate risk ratio (95%CI)	p-value
Surgical outcomes					
Major surgical complications (CD≥3)	6	1.92 (0.41–8.86)	0.395	1.95 (0.43–8.83)	0.385
Reoperation	2	-	-	0.29 (0.12–0.70)	<0.001
Concurrent hysterectomy	31	0.56 (0.28–1.12)	0.076	0.52 (0.07–3.47)	0.500
Predictors for segmental resection					
Defecation pain	11	2.30 (0.77–6.85)	0.124	1.77 (1.20–3.39)	0.014
Rectal bleed	1	-	-	3.00 (2.17–4.13)	0.016
Constipation	5	1.28 (0.23–7.19)	0.777	0.62 (0.17–2.19)	0.419
Any intestinal symptoms	22	1.33 (0.65–2.69)	0.432	1.29 (0.68–2.45)	0.432
Uterine bleed	13	1.20 (0.43–3.30)	0.722	1.11 (0.39–3.13)	0.843
Diarrhea	8	2.02 (1.06–3.05)	0.047	1.99 (1.03–3.82)	0.038

CI: confidence interval; CD: Clavien-Dindo classification.

## DISCUSSION

This study reports the main outcomes of DE patients with intestinal involvement in a tertiary endometriosis referral center. We decided to divide the cohort of patients between the type of intestinal procedure performed (segmental resection group vs. no-colectomy surgery group). The most common overall gynecological symptom found was pelvic pain (68.4%), followed by dysmenorrhea (64.4%), and dyspareunia (48.6%). A study with more than 3,000 patients described an incidence of dysmenorrhea in 95% and dyspareunia in 87%, which can be considered similar to our findings^15^. Referring to intestinal symptoms, our data demonstrated that constipation was the most frequent (18.4%), followed by dyschezia (14.4%), and diarrhea (10.5%). Still, pelvic pain was more frequent in the no-colectomy surgery group and diarrhea was more common in the resection group. The correlation between the severity of symptoms and the disease stage is conflicting. Roman et al. compared three groups of patients related to the extension of the disease and their symptoms (patients with superficial endometriosis, patients with DE, and patients with DE and intestinal involvement). Women presenting with rectal endometriosis were more likely to report an increase in the intensity and length of dysmenorrhea, while deep dyspareunia appeared to be more severe in women with superficial endometriosis. Women reporting rectal endometriosis were more likely to present with cyclic defecation pain (67.9%), cyclic constipation (54.7%), and a significantly longer stool evacuation time. Still, these complaints were also frequent in the other two groups (38.1 and 33.3% in women with superficial endometriosis and 42.9 and 26.2% in women with DE without intestinal involvement, respectively). The authors did not find any independent clinical factors related to infiltration of the rectum by deep endometriosis, and therefore the type of resection^23^. Conversely, our results have shown that dyschezia, rectal bleeding, and above all diarrhea are strong predictive factors for performing RTS.

Most patients with DE of the intestine will require surgery as a definitive treatment at some point. Quality of life (QOL) studies of patients operated on for DE have demonstrated an overall improvement in 85–95% of patients^4^. Several different approaches have been described to treat intestinal lesions depending on the characteristics of the lesions, such as size, percentage of intestinal circumference involvement, depth, and distance from the anal verge^16^. The three most performed surgical techniques are rectal shaving, disc excision, and segmental resection. Whenever possible, it is desirable to avoid segmental resections, especially in lesions close to the anal verge to avoid early complications, such as fistulas, or late complications such as anterior resection syndrome^27^. In our study, the final decision on optimal surgical technique was established intraoperatively.

The overall median size of the largest diameter of intestinal lesions was 2.8 cm. There was a clear difference between the three groups (RTS: 3.1 cm vs. rectal shaving: 1.6 cm vs. disc excision: 2.6 cm; p<0.001), demonstrating that the size of the lesion had a direct impact on the decision of which type of resection was performed. However, this criterion is far from being accepted by other investigators. According to a review by Donnez et al., the size of the nodule should not dictate the type of surgery to be performed^16^. In a study with 63 patients published by Abdalla-Ribeiro et al., they found a cutoff point of 2.25 cm longitudinal lesion size separating the linear nodulectomy from the segmental resection for excising intestinal endometriosis^1^. Similar results were shown by Brey-Beraldo et al., demonstrating that the larger the lesion size, the greater the association with the use of wider intestinal resections (aRR 1.16; 95%CI 1.04–1.30; p=0.007)^11^. Despite that, as in the present study, no correlation was found between lesion size and surgical complications.

Our results have confirmed that no-colectomy surgery offers shorter operative time and length of hospital stay. Obviously, in patients in whom segmental resection could be avoided, the disease was less advanced, and therefore, conservative surgery (less aggressive procedure) could be offered.

Historically, surgical complication rates were relatively higher following segmental resection (RTS) than shaving or disc excision^28^. In a retrospective study with 364 patients (139 treated with RTS), Abo et al. compared the rate of postoperative complications in patients treated by RTS, discoid excision, and rectal shaving. Clavien-Dindo ≥3 complications occurred in 11.8%, and the prevalence of these complications was significantly higher in the RTS group^2^. In another study with 143 patients, of whom 76 were treated with RTS, the rate of postoperative complications was 31.5%. Still, the RTS group had a higher rate of severe postoperative complications in comparison with the disc excision or shaving technique groups (23.5 vs. 5 vs. 0%, respectively)^19^.

It would be expected that postoperative morbidity would be lower in the no-colectomy surgery group. However, our results have shown otherwise. The overall morbidity was 31.5%, but severe complications occurred only in 6 (7.8%) cases. Of these, two-thirds of the patients were in the no-colectomy surgery group. Nevertheless, the multivariate risk ratio regarding major complications between the two groups was not significant (1.95; 95%CI 0.43–8.83; p=0.385). Still, the two patients who required reoperation were in the no-colectomy group (new vaginal dome suturing). Curiously, the no-colectomy surgery group was found to be an independent risk factor for reoperation (0.29; 95%CI 0.12–0.70; p<0.001). In fact, this complication that required a reoperation was more related to the hysterectomy itself rather than any intestinal procedure performed.

Rectovaginal fistula and anastomotic leakage are the two major complications of RTS. Other complications include pelvic abscess, postoperative bleeding, and ureteral damage. A meta-analysis of 3,079 patients published by Balla et al. observed an overall complication rate of 18.5%, and the most frequent complication that occurred was recto-vaginal fistula (2.4%)^5^. Ruffo et al. reported an incidence of ureteral damage between 0.5–3.7% of patients treated with RTS^24^. Although the risk of ureter damage is real, the identification and thorough dissection of both ureters must be carried out to ensure the absence of infiltration by an endometriosis nodule. Extrinsic involvement is treated with “decompression” preferably protected by a double-J stent. Intrinsic ureteral involvement is treated by resection followed by primary anastomosis or ureteral bladder, depending on the distance to the bladder. In our study, extrinsic involvement of the ureter was present in almost 20% of patients, and only one patient required resection. Although the left ureter was more affected than the right, no significant difference was found in ureter involvement.

One of the major risk factors for the occurrence of fistula is the concomitant presence of a suture line from a segmental resection and the excision of vaginal nodules from the vaginal dome after hysterectomy. In our study, there was no anastomotic leakage. One (1.3%) patient developed a rectovaginal fistula on the 9^th^ postoperative day, and another one (1.3%) developed a ureterovaginal fistula on the 10^th^ postoperative day. Both were treated with RTS and concurrent hysterectomy. Even though hysterectomy was not found as an independent risk factor for major complications (0.52; 95%CI 0.07–3.47; p=0.500), one should be alert whenever an associated procedure for the reproductive and urinary organs is necessary. A systematic review reported that about 80% of patients treated with RTS for intestinal endometriosis underwent more than one procedure in the same intervention^5^. For instance, ileocolic locations should be searched carefully intraoperatively because preoperative imaging fails to identify these lesions in over 50% of cases. These may be treated with appendectomy, cecal, ileal or ileocecal resection, depending on the location. In the present study, appendectomy was necessary in 18.5% of patients and enterectomy in 6.5%. Although no significant difference was found, probably due to the low number of patients, the no-colectomy surgery group was responsible for almost 80% of these cases. Bendifallah et al. demonstrated that the occurrence of rectovaginal fistula and anastomotic leakage in centers performing more than 40 procedures per year was 2.8%, compared to 4.9% in centers performing less than ten procedures^8^. This volume-outcome relationship was also identified in other studies for different diseases, whereupon surgical morbidity and mortality have declined considerably due to centralization of patients in high-volume hospitals^6^. Our rate of 6.5% of surgical morbidity and no mortality correspond to outcomes of other high-volume DE centers^5,7^.

Better outcomes tend to be obtained from the first operation when compared to subsequent surgical procedures. Therefore, an excessive number of procedures should be avoided^27^. Vercellini et al. reported that the frequency of Clavien-Dindo ≥3 complications observed in patients undergoing repeated surgery was more than double (14%) of that observed in patients undergoing first-line surgery (6%)^27^. Our data shows that 20% of the patients already had previous surgical treatment, but no increase in the risk of severe complications was found in this group of patients.

Some authors claimed that the risk of rectovaginal fistula or leakage after bowel resection can be reduced when a protective ileostomy at the time of surgery is performed, especially with low anastomosis (<5 cm from the anal verge)^13,26^. In contrast, it has been reported that bowel stenosis occurs in patients who undergo segmental resection, most of them with a diverting stoma, with no cases reported in patients undergoing disc excision, with or without a stoma^10^. The rate of diverting stoma after bowel resection for rectovaginal DE is widely variable, from 1.6 to 96%^17^. In our results, the need of ileostomy was necessary in one (1.3%) case, as a result of failure of the stapling device in a patient undergoing disc excision.

Despite the prospective data collection, our work has the limitations of a retrospective study. For instance, the number of patients enrolled is relatively small and the long-term outcomes such as improvement in quality of life, late complications, and recurrence were not evaluated, and must be the object of future investigations.

## CONCLUSION

Patients with deep endometriosis with intestinal involvement, symptoms like dyschezia, rectal bleeding, and diarrhea during the menstrual period are predictive factors for performing a rectosigmoidectomy. Still, regarding severe complications, no difference was found between patients who underwent segmental resection and those in the no-colectomy surgery group. Surgical treatment of intestinal endometriosis has low morbidity when performed in specialized centers.
